# “A safe, non-judgmental space where I can really challenge myself:” learner experiences in a virtual, case-based diagnostic reasoning conference for students

**DOI:** 10.1080/10872981.2024.2414559

**Published:** 2024-10-14

**Authors:** John C. Penner, María J. Alemán, Andrea Anampa-Guzmán, Aaron L. Berkowitz, Saman Nematollahi

**Affiliations:** a Department of Medicine, University of California San Francisco, San Francisco, CA, USA; b Medical Service, San Francisco VA Medical Center, San Francisco, CA, USA; c School of Medicine, Universidad Francisco Marroquin, Guatemala City, Guatemala; d San Fernando Medical School Faculty of Medicine, Universidad Nacional Mayor de San Marcos, Lima, Peru; e Department of Neurology, University of California, San Francisco, San Francisco, CA, USA; f Division of Infectious Disease, College of Medicine, University of Arizona, Tucson, AZ, USA

**Keywords:** Clinical reasoning, case conferences, morning report, clinical reasoning instruction, clinical teaching

## Abstract

Case-based diagnostic reasoning conferences, like morning reports, allow undergraduate medical trainees to practice diagnostic reasoning alongside senior clinicians. However, trainees have reported discomfort doing so. Peer-assisted learning offers an alternative approach. We describe the design, implementation, and evaluation of a virtual, student-only diagnostic reasoning conference that leverages peer-assisted learning. Student virtual morning report’s (VMR) design was informed by social and cognitive congruence and experience-based learning. We evaluated participant experiences using a survey focused on participant perceptions of Student VMR’s value, their methods for participation, and their preferences for Student VMR compared with VMR with more senior clinicians. 110 participants (28.9%) completed the survey. 90 participants (81.2%) reported that Student VMR was educational. Compared to VMR, participants reported being more likely to participate in Student VMR by turning on their video (50.0%), presenting a case (43.6%), verbally participating (44.5%), or participating in the chat (70.0%). Strengths included a safe learning environment to practice DR and the opportunity to engage with an international learning community. When asked whether they preferred Student VMR or non-Student VMR, most respondents (64.5%, 71/110) identified that they did not have a preference between the two. A student-focused DR conference may offer a valuable complement to, but not a replacement of, apprenticeship-based DR case conferences.

## Introduction

Diagnostic reasoning (DR) education remains an important priority for undergraduate medical education (UME), and several education organizations have recommended its incorporation into medical education curricula to reduce diagnostic error and improve patient safety [1–3]. Despite these recommendations and the development of explicit curricula, limitations to DR instruction in UME persist; many students lack access to structured teaching sessions focused on DR, an understanding of foundational DR concepts, and the ability to apply these concepts to clinical practice [3–7]. While case-based conferences, such as morning reports, are a common venue for explicit DR education, they often focus on graduate medical learners (e.g., residents) [3,8–10]. The intentional design, implementation, and evaluation of case-based DR conferences can help meet the need for improved DR instruction in undergraduate medical curricula.

Case-based DR conferences are a central vehicle for DR instruction and a highly-rated component of clinical education [3,4,8–10]. While pre-clinical curricula increasingly include courses that explicitly focus on teaching DR, case-based conferences are one of the primary sources of targeted DR instruction for UME learners in their clinical years [3,6,11]. These conferences provide a stimulus (e.g., presentation of authentic clinical cases) for developing organized, interconnected knowledge structures that individuals can later use in their day-to-day practice and an opportunity for participants to engage in the cognitive steps of clinical reasoning [4,12–14]. For example, participants (e.g., students, residents, and faculty attendings) have the opportunity to analyze a case sampled from their practice environment, develop or expand diagnostic frameworks and illness scripts, and practice performing and narrating essential elements of DR (e.g., hypothesis generation, problem representation, differential diagnosis, diagnostic justification, and diagnostic testing decisions) [8–10,12–16]. UME learners’ attendance and participation in these conferences alongside more senior clinicians aligns with common models of apprenticeship-based clinical education. This model allows students to learn by engaging in the practice of a relevant activity with support and guidance from those with more experience and expertise [17–20]. In case-based DR conferences, the relevant activity includes reasoning through an authentic clinical case, while guidance and support can stem from observing the thought processes of more experienced clinicians (e.g., residents and faculty in attendance) and interacting with them throughout the case discussion.

Despite the popularity and value of this apprenticeship-based model, recent literature has highlighted its drawbacks [9]. For example, Albert and colleagues identified that some residents, particularly more junior residents, reported discomfort participating in discussions when more senior clinicians (e.g., faculty attendings) are present, citing a lack of psychological safety, as well as concerns about senior clinicians formally evaluating their contributions to the discussion [9]. For UME learners, this discomfort may be exacerbated given their even greater experiential and hierarchical distance from senior residents and attending physicians. Because direct engagement plays an important role in the development of DR capabilities, the presence of more senior clinicians and the consequent decreased learner participation may detract from the educational value of these conferences. Peer-assisted learning offers an alternative model to an apprenticeship-based design and may help overcome the challenges it creates [21–23]. The central premise of this model is that learners can assist their peers in the learning process and, in doing so, also learn themselves [24].

The concepts of social and cognitive congruence underpin the benefits of peer-assisted learning in medical education [23].. The educational value of social congruence stems from the idea that learning from and with social peers (i.e., those with congruent social roles, such as a health professions student) can provide important motivation for learning and foster psychological safety that lessens the barrier to disclosing challenges, confusion, or ignorance of certain pieces of content [22,23,25–27]. Cognitive congruence extends these concepts beyond the social sphere, postulating that learning from and alongside those with similar cognitive structures (e.g., ways of organizing information) will allow for easier and more efficient knowledge expansion [22,23,25,26,28]. Initially proposed as principles to inform learning in primary education, several medical education scholars have highlighted how social and cognitive congruence can enhance learning throughout medical education, particularly with undergraduate health professions trainees [23,25,26,29–31].

Viewing DR education through the lens of peer-assisted learning and social and cognitive congruence suggests that there may be value in creating student-only, case-based DR conferences as a supplement to traditional models in which students learn alongside more senior clinicians. In May 2020, to supplement traditional models of case-based DR conferences, *The Clinical Problem Solvers* (CPSolvers), a multimedia education company focused on creating open-access DR education content, expanded their virtual morning report (VMR) series to include a student-only session (Student VMR) with the goal of creating a psychologically safe case-based conference that offered students the opportunity to practice DR. Here, we describe the design, implementation, and evaluation of Student VMR.

## Materials & methods

### Aims

The aim of Student VMR was to create a psychologically safe case-based DR conference that supports students’ practicing DR.

### Setting & participants

Similar to the structure of other VMR sessions, [10] the CPSolvers hosted Student VMR weekly on Sunday mornings (PST) on a video conferencing platform (Zoom Video Communications Inc., San Jose, CA, USA). Student VMR sessions operate independently from any university affiliation. Participants included health professions students who asked to attend Student VMR and confirmed their enrollment in a health professions education program, which allowed them to join the Student VMR email listserv. Instructions for joining sessions were distributed via the listserv each week.

### Program design

The design of Student VMR was informed by the benefits of learning in the setting of social and cognitive congruence, and by our prior design of VMR, which draws on Dornan et al.’s Experience-Based Learning model (ExBL) [10,17,22,23].

#### Rationale for a student-only conference: social and cognitive congruence

As co-designers, facilitators, and participants of CPSolvers’ VMR, we received informal feedback from student participants that they felt uncomfortable and intimidated participating in VMR alongside experienced graduate trainees and faculty clinicians. We related this feedback to the literature on the value of peer-assisted learning in medical education and the roles of social and cognitive congruence, as well as prior work that has identified how the presence of senior clinicians can detract from more junior participants’ comfort contributing to case discussions [9,22,23]. We aimed to address this feedback by intentionally creating a learning environment structured for social and cognitive *congruence*.

#### Conference structure, teaching strategies, & participation opportunities

**Figure 1.**
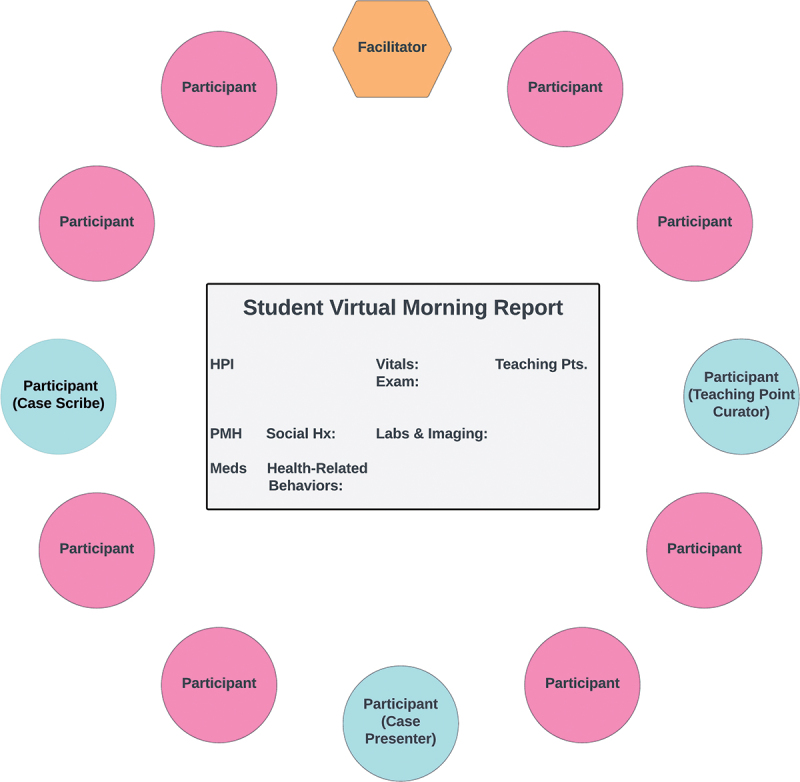
A conceptual diagram of the structure of Student VMR. Roles include the facilitator (hexagon), who deliberately promoted peer-assisted clinical reasoning learning, and participants (circles). Certain participants had specific roles: case presenter (sharing sequential aliquots of a case), case scribe (transcribing case details onto the virtual whiteboard), and teaching point curator (transcribing participant-generated teaching points and summarizing them at the end of the session).

Student VMR’s structure is similar to that of conventional morning reports in United States residency programs (Figure 1) [8,9,32–34]. The full description of the VMR structure has been previously described [10]. Here, we highlight salient components of the design. One participant volunteers to present a case in sequential aliquots of clinical data, followed by a final diagnosis. To promote real-time clinical problem-solving, Student VMR is unscripted, meaning no participants other than the case presenter are aware of the case details. After each aliquot of information, the facilitator asks probing questions of the group to catalyze their reasoning and promote their engagement with and teaching of one another. In other words, the facilitator deliberately promotes peer-assisted DR learning, anchoring these questions to cognitive elements of DR (e.g., problem representation, diagnostic frameworks, illness scripts, and Bayesian reasoning) and contextual factors (e.g., resource availability, patient communication, and interactions with other healthcare team members) [35–38]. Participants then share their reasoning verbally or via the text-based chat function, and the facilitator offers summarizing reflections, highlights points shared in the chat, or, occasionally, adds additional perspectives that may support participants’ reasoning before the next aliquot.

While the facilitator’s presence held the potential to disrupt social and cognitive congruence, we believe several important factors in facilitator selection mitigated this: the facilitator (1) was a medical resident or chief medical resident (i.e., someone with more relative social and cognitive congruence than an experienced faculty member), (2) was consistent across the sessions, allowing for relationship building and participant comfort, and (3) did not perform learner assessments given Student VMR’s independence from formal curricula.

As previously described in our prior study of VMR, Dornan et al.’s Experience-Based Learning model (ExBL) informed the design of opportunities for participation [10,17]. This conceptual framework emphasizes the value of a variety of opportunities for supported engagement in a specific practice. In Student VMR, participation involves practicing and narrating DR via text-based or verbal articulation, with support coming from near-peers and, occasionally, the facilitator. In addition, participants have the option of engaging by presenting a case, scribing the case on the virtual whiteboard, or curating teaching points.

### Program evaluation

We evaluated Student VMR by developing a survey with both open and closed-ended questions following previously described guidelines [39]. Survey items (Appendix 1) focused on participant perceptions of the educational value of Student VMR, their overall experience in Student VMR, their methods for participation in Student VMR, and their preferences for Student VMR compared with VMR (e.g., with more senior clinicians).

We gathered validity evidence related to content and response process [40]. We piloted survey items with five CPSolvers team members who had participated in Student VMR lessons. In addition, we completed cognitive interviews with six student VMR participants who volunteered to participate in these interviews prior to survey distribution. Participants were recruited from Student VMR sessions. Given Student VMR’s international participants, we included individuals who do not speak English as their first language to ensure we corrected any potential areas of misunderstandings prior to distribution. One team member with cognitive interviewing experience (M.J.A.) used previously published guidelines to conduct the interviews and focused interview questions on participants’ comprehension of survey items [40]. Pilot participants’ and cognitive interviewees’ verbal feedback led to changes in item wording for improved question clarity.

We created and administered the survey using Qualtrics and invited participation via email. Individuals who had expressed interest in attending Student VMR by signing up for the student VMR email listserv were eligible to take the survey (*n* = 381). Participants received a link to the survey via email. Data collection occurred between 11 January 2022, and 21 May 2022. To maximize the response rate, we sent weekly reminders for the first six weeks. No financial incentives were offered. All responses were anonymous. The Institutional Review Board at the University of California, San Francisco reviewed the study and deemed it exempt (IRB reference #296328).

Two coders (J.C.P. & S.N.) used an iterative, constant-comparative approach to thematic analysis to manually code free-response data and identify themes [41]. They discussed and resolved conflicts together.

## Results

All three hundred eighty-one students on the email listserv for Student VMR received the survey invitation. One hundred ten participants (28.9%, 110/381) completed the survey. Demographic information is summarized in Table 1. Ninety (81.2%, 90/110) participants reported that Student VMR was very educational or educational (Table 2). Compared to VMR, many participants reported being more likely to participate in Student VMR by turning on their video (*n* = 55, 50.0%), presenting a case (43.6%), participating verbally in the case discussion (44.5%), or participating in the chat (70.0%) (Table 2).

**Table 1.** Participant demographics.

Participants’ Self-Identified Gender (*n* = 110)	Countries Represented (*n* = 18)
Man: 49 (44.5%) Woman: 29 (26.3%) Non-binary: 1 (0.9%) Did not disclose: 31 (28.2%)	Argentina: 1 Austria: 3 Brazil: 10 Bulgaria: 1 Cameroon: 1 Canada: 7 Germany: 1 Guatemala: 3 Honduras: 1 India: 9 Iran: 1 Nigeria: 1 Peru: 1 Qatar: 1 Saudi Arabia: 1 Somalia: 1 United States of America: 28 Vietnam: 1 Did not disclose: 38
Participants’ Self-Identified Race/Ethnicity (*n* = 110)	
White: 19 (17.2%) Asian: 21 (19.1%) Black: 10 (9.1%) Hispanic/LatinX: 23 (20.9%) Prefer to self-describe: 4 (3.6%) African: 2 (1.8%) Middle Eastern: 2 (1.8%) Did not disclose: 33 (30.0%)

**Table 2.** Quantitative data on participant perceptions of the value of Student VMR and their participation preferences.

Educational Value of Student VMR (*n* = 110)			Preference of Student VMR or Non-Student VMR		
Very educational75 (68.2%) Educational15 (13.6%) Neutral0 (0.0%) Non-educational0 (0.0%) Very non-educational0 (0.0%) Did not answer20 (18.1%)					
			I prefer Student VMR: 10 (9.1%) I prefer Non-Student VMR: 10 (9.1%) No preference: 71 (64.5%) Did not answer 19 (17.3%)		
Likelihood of participation in Student VMR compared to non-Student VMR (*n* = 110)					
Form of Participation	More likely	Neither more nor less likely		Less likely	Did not answer
Turn on video	55 (50.0%)	21 (19.1%)		14 (12.7%)	20 (18.2%)
Present a case	48 (43.6%)	28 (25.5%)		12 (10.9%)	22 (20.0%)
Verbally participate in the discussion	49 (44.5%)	24 (21.8%)		16 (14.5%)	21 (19.1%)
Participate via chat	77 (70.0%)	10 (9.0%)		3 (2.7%)	20 (18.2%)

Thematic analysis of free-text responses identified two themes regarding the strengths of Student VMR: (1) Student VMR provides a safe learning environment to practice DR amongst fellow students and (2) Student VMR provides the opportunity to broaden perspectives amidst an international learning community (Table 3). Participants reported that the contributors to a safe learning environment to practice DR included participants’ sense of psychological safety learning alongside near-peers, engagement in case-based learning, and perception that the educational content aligned with their level of knowledge. The international learning community allowed participants to learn about clinical diseases they may not come across in their local context, facilitated new connections with near peers in the learning community of Student VMR, and, for participants for whom English was not their first language, provided opportunities to practice medical English.

**Table 3.** Qualitative data regarding strengths of student VMR, barriers to participation, and reasons for preferring both student and non-student VMR.

Theme	Representative Quotes
**Strengths of Student VMR**	
A safe learning environment to practice clinical reasoning amongst fellow students	*Student VMR is a safe non-judgmental space where I can test out my ideas and really challenge myself and also connect with other students from all around the world.* *There is more time for more detailed explanations for diagnostic thinking in student VMR. Less pressure.* *Most of us are students so it’s nice to see people in similar situations experience diagnostic reasoning and we can all learn together.*
Student VMR provides the opportunity to broaden perspectives amidst an international learning community	*The diversity of the backgrounds of the participants is amazing.* *Practice my English skills (am not native speaker).* *Love the international community; so many different perspectives that I would not be exposed to without the virtual option.* *Diverse clinical presentations from various epidemiologies of the world help us broaden our scope of thinking.*
**Barriers to Participation in Student VMR**	
Individual factors	*I am camera shy and have social anxiety.* *Thinking that I don’t have enough knowledge to discuss.* *Its rhythm is quite fast (as english is not my mother tongue) and I am not used with the medical terms used.*
Contextual factors	*My internet connection, which in this part of the world is poor.* *It’s usually, the time zone constraint. Most of the time the VMRs are late night or early mornings for me … I’m tired or just not in the headspace to discuss after a day of work.* *I am working on weekends. Unfortunately, I can not open my microphone or interact with my camera.*
**Reasons for Preferring Both Student VMR and Non-Student VMR**	
The complementary nature of participating in clinical reasoning conferences informed by peer-assisted learning and those informed by apprenticeship-based learning.	*Student VMR: learning the basic knowledge. Non student VMR: learn and practice advanced clinical skill and knowledge.* *I love in student VMR the slow pace. In non student VMR I love to see how the clinicians think. I actually love both and get so much knowledge that I lack.* *I think that both opportunities provide different emphasis on an educational basis. Student VMR allowed us to share with peers (in case you’re still in school) techniques that somehow will relate to our current situation. And a non-student VMR gives us the chance of taking a glimpse of the clinical reasoning process followed by experts.*

Barriers to participation arose from individual and contextual factors. Individual factors included self-reported shyness, lack of confidence in their clinical knowledge, fear of being wrong, and language discordance (e.g., individuals for whom English was not their first language reported apprehension and difficulty contributing to the discussion). Contextual factors related to learners’ location (e.g., time zone), technological limitations (e.g., internet bandwidth connectivity), access to clinical cases to present, and competing obligations (e.g., clinical work occurring at the same time) (Table 3).

When asked whether they preferred Student VMR or non-Student VMR, or did not have a preference, most respondents (64.5%, 71/110) identified that they did not have a preference between the two. Free responses elaborating on this topic described the complementary nature of participating in DR conferences with only near-peers and in those with more senior clinicians (e.g., residents and faculty) (Table 3). Participants viewed Student VMR’s slow, deliberate discussions of foundational DR knowledge and VMR’s faster, more complex case discussions as mutually supportive. They also emphasized the importance of both learning alongside near-peers, because of the relatedness and belonging it affords, and learning from and with experts, because of the exposure to advanced DR it offers.

## Discussion

While DR instruction remains a key component of health professions education curricula, UME learners have variable access to educational experiences that facilitate the development of DR abilities, such as case-based teaching conferences [2,3]. Furthermore, the common structure of these conferences, which is often based on apprenticeship learning models, can limit more junior learners’ participation due to discomfort sharing their thoughts in front of more senior clinicians [9]. In this study, we show that participants perceived a student-focused case-based DR conference to have offered educational value and facilitated increased engagement and participation. In addition, participant responses suggested that the presence of social and cognitive congruence supported their perceptions of the conference’s educational value and their likelihood of participation. Finally, this study suggests that student-focused, case-based clinical-reasoning conferences may offer a valuable complement to, but not a replacement of, the common apprenticeship-based model of DR case conferences.

Participants reported a higher likelihood of engaging in Student VMR (e.g., by turning on their video, presenting a case, or contributing to the case discussion) compared to non-Student VMR, which includes consistent attendance by more senior clinicians (e.g., residents and faculty). Students’ increased likelihood of participating in the absence of more senior clinicians aligns with work by Albert and colleagues exploring internal medicine residents’ perceptions of morning report [9]. They discovered that 1st year residents were the most likely to feel discomfort contributing to the case discussion and identified that a fear of being wrong, perceptions of the safety and inclusivity in the learning environment, and the presence or absence of attending physicians influenced this discomfort. Our study adds to the literature regarding the role of the learning environment in case-based DR conferences. The fact that participants frequently emphasized their comfort engaging in DR alongside near peers suggests that a student-focused session can contribute to the psychological safety necessary for participation and aligns with prior work on the value of social congruence in peer-assisted learning experiences [22,30,31].

Consistent with prior studies of open-access, virtual case-based DR conferences, participants also highlighted the value of Student VMR’s international learning community [10,42]. These benefits include getting exposure to cases they may not see in their local context (e.g., a participant in Germany learning from a case of visceral Leishmaniasis presented by a student from Brazil; a participant from Morocco learning from a case of coccidioidomycosis presented by a student in the western United States). For those students for whom English is not their first language, Student VMR also offered opportunities to learn and practice medical English. Given the perceived advantages of an international learning community and the opportunities provided by technological advancements in delivering effective case-based teaching through virtual platforms, we encourage health professions educators with established platforms for case-based DR conferences to explore avenues for extending virtual access to international audiences. This may be particularly impactful for learners from low- and middle-income countries, many of whom face disproportionately large barriers to accessing DR education [43].

However, even in the presence of near-peers, some participants still expressed fears of being wrong and a lack of confidence as a barrier to participation. Indeed, contributing to a case-based DR discussion requires learners to make themselves vulnerable for the sake of growth. Exposing one’s thought processes, voicing uncertainties, and opening up to new ways of understanding engenders risks, such as a loss of credibility or ‘face’ (e.g., ‘one’s image of self, delineated in terms of approved social attributes’) in front of colleagues [44,45]. These ‘risky’ moments abound in case-based DR discussions, during which learners share their understanding – or misunderstanding- of key elements of clinical practice and justify clinical decisions they may make in certain scenarios. We encourage educators to continue to explore ways to explicitly promote a culture of what Bearman and Molloy have termed ‘intellectual candor’ in case-based DR conferences [45]. Possible strategies include elevating and celebrating when learners reveal unpolished thought processes, expose areas of limited understanding, or disclose errors or mistakes they may have made in similar cases [45,46].

Participants also perceived the discussions in Student VMR to be more effectively tailored to their learning needs compared to VMR with more advanced clinicians. This aligns with prior work on cognitive congruence [23,24,28] and relates to Vygotsky’s zone of proximal development (ZPD), or the gap between a learner’s capability and their currently accessible developmental potential. According to Vygotsky, optimal learning occurs when one engages with material within their ZPD alongside others [47,48]. Engaging in DR with near peers (e.g., those with cognitive congruence) may help learners more easily access and explore their ZPDs together. Given the importance of knowledge structures (e.g., diagnostic frameworks and illness scripts) in DR, cognitive congruence may play a particularly valuable role in developing the content knowledge necessary for clinical practice [14,16]. Future studies exploring the role of cognitive congruence in DR education may provide useful insights into pedagogical strategies that can promote the development of DR capabilities.

Finally, rather than preferring student-focused, case-based DR conferences or those that include more senior clinicians, participants perceived unique value from both formats. We believe this highlights an important principle regarding the development of DR curricula: there is value in creating opportunities for learners to engage in DR alongside near-peers *and* alongside senior clinicians with more experience and expertise. The combination of peer-assisted and apprenticeship-based education models is prominent throughout health professions education curricula, though the literature on the combination of the two is less robust in the clinical environment where case-based DR conferences often take place [6,21,23,29,49,50].

Limitations to this study include a low response rate, which may be due to the distribution of the survey on an email list that includes individuals who are not students (and thus ineligible to participate in Student VMR), and those who had expressed interest in student VMR but never participated. The subjective responses regarding participation are based on student perceptions, which limits our ability to objectively assess whether individuals are actually participating more in Student VMR than in non-Student VMR.

A student-focused, case-based DR conference informed by principles of peer-assisted learning is a viable strategy for delivering DR instruction and may support increased participation from health professions students. Creating opportunities for student-only case-based DR conferences can leverage the benefits of social and cognitive congruence in peer-assisted learning and offer a valuable complement to, but not a replacement of, apprenticeship-based DR instruction.

## Appendix #1: Survey Questions

1. Do you consent to participate in this research project by taking this survey?

o Yes

o No (please stop the survey here if you do not consent to participate)

2. Have you ever attended Student VMR?

o Yes

o No

3. Overall, how satisfied are you with Student VMR?

o Extremely satisfied

o Somewhat satisfied

o Neither satisfied nor dissatisfied

o Somewhat dissatisfied

o Extremely dissatisfied

4. Compared to non-Student VMR, how likely are you to do each of the following during Student VMR?

**Table:**

	Likely	Neither likely nor unlikely	Unlikely
Turn on your video	o	o	o
Volunteer to present a case	o	o	o
Volunteer to discuss a case	o	o	o
Participate in the Zoom chat	o	o	o

5. How would you describe the educational value of Student VMR?

o  Very educational

o  Educational

o  Neither educational nor non-educational

o  Non-educational

o  Very non-educational

6. Which of the following do you view as a strength of Student VMR? (Choose all that apply)

▢ It gives me access to clinical reasoning experts at institutions other than my own

▢ It is a safe learning environment

▢ Health care provider role modeling

▢ I get to practice my clinical reasoning

▢ I get to connect with other people I would not normally connect with

▢ Other (please elaborate below) __________________________________________________

7. Which skillset have you gained from attending Student VMR? (Choose all that apply)

▢ Diagnostic reasoning

▢ Learning medical content

▢ Becoming a better educator

▢ Networking

▢ Community building

▢ Other (please elaborate below) __________________________________________________

8. Do you prefer Student VMR or non-student VMR?

▢ Student VMR

▢ Non-student VMR

▢ Both

9. Why do you prefer student VMR or non-student VMR?

______________________________________________________________________________________________________________________________________________________________________________________________________________________________________________________________________________________________________________________________________________________________

10. What, if anything, prevents you from **discussing** a case during Student VMR?

_____________________________________________________________________________________________________________________________________________________________________________________________________________________________________________________________________________________________________________________________________________________________________________________________________

11. What, if anything, prevents you from **presenting** a case during Student VMR?

_____________________________________________________________________________________________________________________________________________________________________________________________________________________________________________________________________________________________________________________________________________________________________________________________________

12. What, if anything, prevents you from turning on your video/camera?

_______________________________________________________________________________________________________________________________________________________________________________________________________________________________________________________________________________________________________________________________________________________________________________________________________

13. What else would you like to share about Student VMR?

_______________________________________________________________________________________________________________________________________________________________________________________________________________________________________________________________________________________________________________________________________________________________________________________________________

## References

[1] Committee on Diagnostic Error in Health Care. Board on health care services, institute of medicine, the national academies of sciences, engineering, and medicine. In: Balogh E, Miller B, Ball J, editors. Improving diagnosis in health care. National Academies Press; 2015. p. 21794. doi: 10.17226/21794 26803862

[2] Connor DM, Durning SJ, Rencic JJ. Clinical reasoning as a core competency. Academic Med. 2020;95(8):1166–11. doi: 10.1097/ACM.0000000000003027 31577583

[3] Rencic J, Trowbridge RL, Fagan M, et al. Clinical reasoning education at US medical schools: results from a national survey of internal medicine clerkship directors. J Gen Intern Med. 2017;32(11):1242–1246. doi: 10.1007/s11606-017-4159-y 28840454 PMC5653563

[4] Ten Cate O, Custers EJFM, Durning SJ, editors. Principles and practice of case-based clinical reasoning education. Vol. 15. Springer International Publishing; 2018. doi: 10.1007/978-3-319-64828-6 31314234

[5] Jacobson K, Fisher DL, Hoffman K, et al. Integrated cases section: a course designed to promote clinical reasoning in year 2 medical students. Teach Learn Med. 2010;22(4):312–316. doi: 10.1080/10401334.2010.512835 20936581

[6] Connor DM, Narayana S, Dhaliwal G. A clinical reasoning curriculum for medical students: an interim analysis. Diagnosis. 2022;9(2):265–273. doi: 10.1515/dx-2021-0112 34904425

[7] Lee A, Joynt GM, Lee AKT, et al. Using illness scripts to teach clinical reasoning skills to medical students. Fam Med. 2010;42(4):255–261. 20373168

[8] Amin Z, Guajardo J, Wisniewski W, et al. Morning report: focus and methods over the past three decades. Acad Med. 2000;75(10 Suppl):S1–5. doi: 10.1097/00001888-200010001-00002 11031158

[9] Albert TJ, Redinger J, Starks H, et al. Internal medicine residents’ perceptions of morning report: a multicenter survey. J Gen Intern Med. [cited 2021 Jan 14];36(3):647–653. doi: 10.1007/s11606-020-06351-7 33443704 PMC7947099

[10] Penner JC, Le S, Shipley LC, et al. Morning report goes virtual: learner experiences in a virtual, case-based diagnostic reasoning conference. Diagnosis. 2022;9(1):89–95. doi: 10.1515/dx-2021-0073 34348421

[11] Hawks MK, Maciuba JM, Merkebu J, et al. Clinical reasoning curricula in preclinical undergraduate medical education: a scoping review. Academic Med. 2023;98(8):958–965. doi: 10.1097/ACM.0000000000005197 36862627

[12] Eva KW. What every teacher needs to know about clinical reasoning. Med Educ. 2005;39(1):98–106. doi: 10.1111/j.1365-2929.2004.01972.x 15612906

[13] Norman G. Research in clinical reasoning: past history and current trends. Med Educ. 2005;39(4):418–427. doi: 10.1111/j.1365-2929.2005.02127.x 15813765

[14] Elstein AS, Schwartz A. Clinical problem solving and diagnostic decision making: selective review of the cognitive literature. BMJ. 2002;324(7339):729–732. doi: 10.1136/bmj.324.7339.729 11909793 PMC1122649

[15] Heppe DB, Beard AS, Cornia PB, et al. A multicenter VA study of the format and content of internal medicine morning report. J Gen Intern Med. 2020;35(12):3591–3596. doi: 10.1007/s11606-020-06069-6 32779143 PMC7728907

[16] Custers EJFM. Thirty years of illness scripts: theoretical origins and practical applications. Med Teach. 2015;37(5):457–462. doi: 10.3109/0142159X.2014.956052 25180878

[17] Dornan T, Conn R, Monaghan H, et al. Experience based learning (ExBL): clinical teaching for the twenty-first century. Med Teach. 2019;41(10):1098–1105. doi: 10.1080/0142159X.2019.1630730 31382787

[18] Teunissen PW, Scheele F, Scherpbier AJJA, et al. How residents learn: qualitative evidence for the pivotal role of clinical activities. Med Educ. 2007;41(8):763–770. doi: 10.1111/j.1365-2923.2007.02778.x 17661884

[19] Stalmeijer RE, Dolmans DHJM, Wolfhagen IHAP, et al. Cognitive apprenticeship in clinical practice: can it stimulate learning in the opinion of students? Adv Health Sci Educ. 2009;14(4):535–546. doi: 10.1007/s10459-008-9136-0 PMC2744784 18798005

[20] Stalmeijer RE, Dolmans DHJM, Snellen-Balendong HAM, et al. Clinical teaching based on principles of cognitive apprenticeship: views of experienced clinical teachers. Academic Med. 2013;88(6):861–865. doi: 10.1097/ACM.0b013e31828fff12 23619074

[21] Moore-West M, Hennessy SA, Meilman PW, et al. The presence of student-based peer advising, peer tutoring, and performance evaluation programs among U.S. medical schools. Acad Med. 1990;65(10):660–661. doi: 10.1097/00001888-199010000-00018 2261049

[22] Ten Cate O, Durning S. Dimensions and psychology of peer teaching in medical education. Med Teach. 2007;29(6):546–552. doi: 10.1080/01421590701583816 17978967

[23] Lockspeiser TM, O’Sullivan P, Teherani A, et al. Understanding the experience of being taught by peers: the value of social and cognitive congruence. Adv Health Sci Educ Theory Pract. 2008;13(3):361–372. doi: 10.1007/s10459-006-9049-8 17124627

[24] Topping KJ. The effectiveness of peer tutoring in further and higher education: a typology and review of the literature. High Educ. 1996;32(3):321–345. doi: 10.1007/BF00138870

[25] Schmidt HG, Moust JH. What makes a tutor effective? A structural-equations modeling approach to learning in problem-based curricula. Acad Med. 1995;70(8):708–714. doi: 10.1097/00001888-199508000-00015 7646747

[26] Moust JHC, Schmidt HG. Facilitating small-group learning: a comparison of student and staff tutors’ behavior. Instr Sci. 1995;22(4):287–301. doi: 10.1007/BF00891782

[27] Sarbin TR. Cross-age tutoring and social identity. In: Children As Teachers. Elsevier; 1976. p. 27–40. doi: 10.1016/B978-0-12-052640-6.50008-X

[28] Cornwall MG. Students as teachers: peer teaching in higher education. Amsterdam, The Netherlands: Universiteit; 1976.

[29] Loda T, Erschens R, Loenneker H, et al. Cognitive and social congruence in peer-assisted learning – a scoping review. PLOS ONE. 2019;14(9):e0222224. doi: 10.1371/journal.pone.0222224 31498826 PMC6733464

[30] Chou CL, Teherani A. A foundation for vital academic and social support in clerkships: learning through peer continuity. Acad Med. 2017;92(7):951–955. doi: 10.1097/ACM.0000000000001661 28353506

[31] Tai J, Penman M, Chou C, et al. Learning with and from peers in clinical education. In: Nestel D, Reedy G, McKenna L Gough S, editors. Clinical education for the health professions. Vol. 2021. Springer Singapore. p. 1–19. doi: 10.1007/978-981-13-6106-7_90-1

[32] Layne K, Nabeebaccus A, Fok H, et al. Modernising morning report: innovation in teaching and learning. Clin Teach. 2010;7(2):77–82. doi: 10.1111/j.1743-498X.2010.00357.x 21134153

[33] Parrino TA. The social transformation of medical morning report. J Gen Intern Med. 1997;12(5):332–333. doi: 10.1046/j.1525-1497.1997.012005332.x 9159704 PMC1497114

[34] Lessing JN, Wheeler DJ, Beaman J, et al. How to facilitate an unscripted morning report case conference. Clin Teach. 2020;17(4):360–365. doi: 10.1111/tct.13111 31749299

[35] Merkebu J, Battistone M, McMains K, et al. Situativity: a family of social cognitive theories for understanding clinical reasoning and diagnostic error. Diagnosis (Berl). 2020;7(3):169–176. doi: 10.1515/dx-2019-0100 32924378

[36] Torre D, Durning SJ, Rencic J, et al. Widening the lens on teaching and assessing clinical reasoning: from “in the head” to “out in the world. Diagnosis (Berl). 2020;7(3):181–190. doi: 10.1515/dx-2019-0098 32142479

[37] ten Cate O, Durning SJ. Understanding clinical reasoning from multiple perspectives: a conceptual and theoretical overview. In: Ten Cate O, Custers E Durning S, editors. Principles and practice of case-based clinical reasoning education: a method for preclinical students. Springer; 2018 [cited 2021 Jan 26]. Available from: http://www.ncbi.nlm.nih.gov/books/NBK543757/

[38] Durning SJ, Artino AR. Situativity theory: a perspective on how participants and the environment can interact: AMEE Guide no. 52. Med Teach. 2011;33(3):188–199. doi: 10.3109/0142159X.2011.550965 21345059

[39] Creswell JW, Plano Clark VL. Designing and conducting mixed methods research. Third ed. Thousand Oaks, CA, USA: SAGE; 2018.

[40] Artino AR, La Rochelle JS, Dezee KJ, et al. Developing questionnaires for educational research: AMEE guide no. 87. Med Teach. 2014;36(6):463–474. doi: 10.3109/0142159X.2014.889814 24661014 PMC4059192

[41] Braun V, Clarke V. Using thematic analysis in psychology. Qualitative Res Phychol. 2006;3(2):77–101. doi: 10.1191/1478088706qp063oa

[42] Lau YTK, Alemán MJ, Medina R, et al. Around the world in 60 minutes: how a virtual morning report has created an international community for clinical reasoning and medical education. Teach Learn Med, 2024;36(3):1–10. Published online June 21. doi: 10.1080/10401334.2023.2226661 37341557

[43] Posever N, Sehdev M, Sylla M, et al. Addressing equity in global medical education during the COVID-19 pandemic: the global medical education collaborative. Acad Med. 2021;96(11):1574–1579. doi: 10.1097/ACM.0000000000004230 34261867 PMC8541891

[44] Goffman E. The presentation of self in everyday life. 1 ed. New York, NY: Anchor Books ed. rev. ed. Anchor Books; 1990.

[45] Molloy E, Bearman M. Embracing the tension between vulnerability and credibility: ‘intellectual candour’ in health professions education. Med Educ. 2019;53(1):32–41. doi: 10.1111/medu.13649 30192024

[46] Jagannath AD, Dreicer JJ, Penner JC, et al. The cognitive apprenticeship: advancing reasoning education by thinking aloud. Diagnosis (Berl). [cited 2022 Dec 1];10(1):9–12. doi: 10.1515/dx-2022-0043 36450097

[47] Topping KJ. Trends in peer learning. Educ Phychol. 2005;25(6):631–645. doi: 10.1080/01443410500345172

[48] Vygotsky LS. Mind in society: development of higher psychological processes. Cole M, Jolm-Steiner V, Scribner S, et al., editors. Harvard University Press; 1980. doi: 10.2307/j.ctvjf9vz4

[49] Tai J, Molloy E, Haines T, et al. Same-level peer-assisted learning in medical clinical placements: a narrative systematic review. Med Educ. 2016;50(4):469–484. doi: 10.1111/medu.12898 26995485

[50] Ten Cate O, Durning S. Peer teaching in medical education: twelve reasons to move from theory to practice. Med Teach. 2007;29(6):591–599. doi: 10.1080/01421590701606799 17922354

